# The Effects of Blue Light on Human Fibroblasts and Diabetic Wound Healing

**DOI:** 10.3390/life12091431

**Published:** 2022-09-14

**Authors:** Meesha Purbhoo-Makan, Nicolette Nadene Houreld, Chukuka S. Enwemeka

**Affiliations:** 1 Department of Podiatry, Faculty of Health Sciences, University of Johannesburg, P.O. Box 17011, Doornfontein 2028, South Africa; 2 Laser Research Center, Faculty of Health Sciences, University of Johannesburg, P.O. Box 17011, Doornfontein 2028, South Africa; 3 College of Health and Human Services, San Diego State University, San Diego, CA 92182, USA

**Keywords:** antibiotic resistance, diabetic foot ulcers, fibroblast proliferation, photobiomodulation, wound healing

## Abstract

Diabetes is a serious threat to global health and is among the top 10 causes of death. The Diabetic foot ulcer (DFU) is among the most common and severe complications of the disease. Bacterial infections are common; therefore, timely aggressive management, using multidisciplinary management approaches is needed to prevent complications, morbidity, and mortality, particularly in view of the growing cases of antibiotic-resistant bacteria. Photobiomodulation (PBM) involves the application of low-level light at specific wavelengths to induce cellular photochemical and photophysical responses. Red and near-infrared (NIR) wavelengths have been shown to be beneficial, and recent studies indicate that other wavelengths within the visible spectrum could be helpful as well, including blue light (400–500 nm). Reports of the antimicrobial activity and susceptibility of blue light on several strains of the same bacterium show that many bacteria are less likely to develop resistance to blue light treatment, meaning it is a viable alternative to antibiotic therapy. However, not all studies have shown positive results for wound healing and fibroblast proliferation. This paper presents a critical review of the literature concerning the use of PBM, with a focus on blue light, for tissue healing and diabetic ulcer care, identifies the pros and cons of PBM intervention, and recommends the potential role of PBM for diabetic ulcer care.

## 1. Introduction

**Figure 1:**
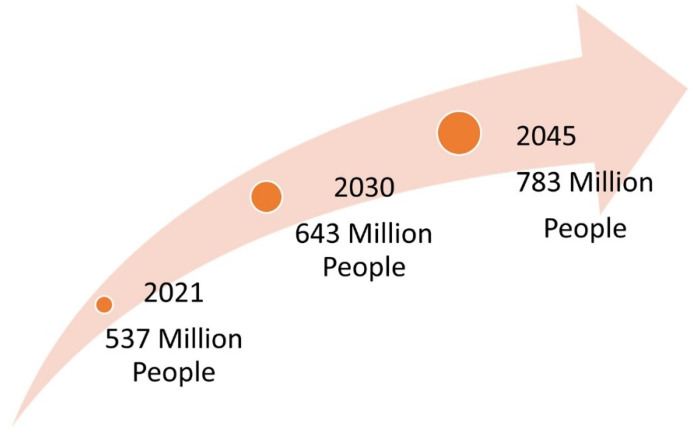
Estimated global predictions of diabetes mellitus.

Diabetes has been reported as a serious threat to global health with no socioeconomic or national boundaries, meaning it is among the top 10 causes of death. With no cure, it is one of the fastest-growing global pandemics of the 21st century (Figure 1). In 2021, global predictions indicated that 537 million people have been diagnosed with diabetes and that these numbers will increase to 643 million by 2030 and 783 million by 2045 [1]. The African continent has the highest number of undiagnosed cases, with more than half of the population (53.6%) not knowing their diabetic status [1]. In South Africa, there was a 9.2% self-reported prevalence of diabetes, which increased with age [2]. Predictions have indicated that South Africa has the highest prevalence of diabetes (4.2 million) and diabetes-attributed mortality rate (95,676) in Africa [1].

**Figure 2:**
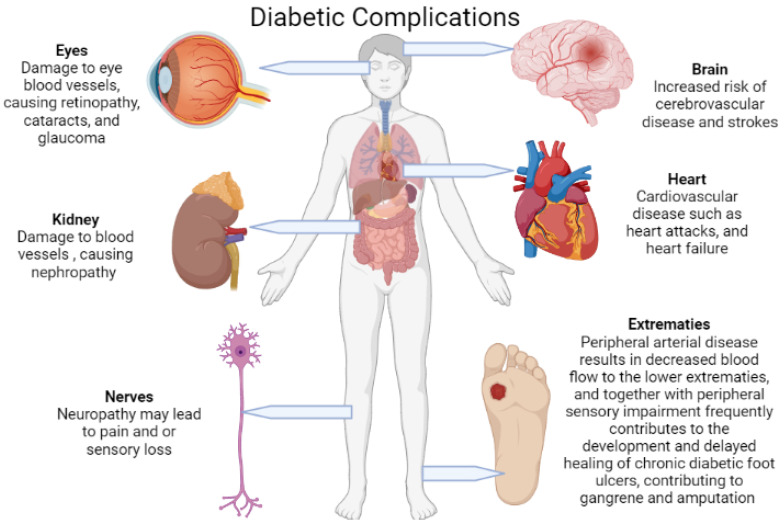
Diabetes mellitus causes several serious and life-threatening complications due to microvascular and macrovascular damage. This damage in turn leads to pathologies mainly in the brain, heart, eyes, kidneys, nerves, and lower extremities (created with BioRender).

Diabetes mellitus (DM) is the cause of several long-term, potentially life-threatening risks and severe complications (Figure 2). These include cardiovascular disease, neuropathy, nephropathy, retinopathy, and diabetic foot ulcers (DFUs), all of which are liable for a substantial increase in morbidity and mortality, particularly when not well managed [2,3,4]. Peripheral arterial disease (PAD) and peripheral sensory impairment are the main attributors to the development of DFUs, which are further complicated by an amalgam of foot deformities and diabetic foot infections (DFIs). These infections span from superficial cellulitis to chronic osteomyelitis which ultimately leads to gangrenous extremity lower limb amputations [4,5,6,7,8]. Foot infections and amputations are among the most frequent and severe complications of DFUs [3,5,6,7,8].

Chronic, severe, and slow-healing wounds are known to have a negative impact on morbidity rates, psychosocial wellness, and financial status globally [9]. Consequently, 23% of Africa’s total health budget is spent on diabetes, meaning it is the largest percentage of health expenditure [3]. Medical literature on the complications of the diabetic foot and public health-related aspects and implications in the African continent is lacking [10]. Due to the wide unavailability of medical insurance or reimbursement of medical expenses in Africa, the treatment and management of a DFU could cost a patient an average income equivalent to 2 years, as was seen in a study in Tanzania [10]. The loss of a lower limb due to complications that arise in the treatment and management, or lack thereof, of DFUs not only places a financial burden on patients, their families, and the government but also places a huge psychological strain on patients and affects their quality of life due to disability, which further impacts on their livelihood.

Diabetic foot and lower limb complications affect 40 to 60 million diabetic patients globally and it is the principal cause of morbidity in diabetic patients [3]. It is estimated that 25% of all diabetic patients will develop a DFU [11], and worldwide, a lower limb or part thereof is lost every 30 seconds due to DM [12]. The 5-year survival rate for an amputee is 45–55% [13,14], and the risk of mortality at ten years is twice as compared to those who do not have diabetes [15]. The treatment and management of DFUs with an infective aetiology further complicate the matter.

Antibiotic therapy has led to a decline in mortality and lower limb amputations; however, the rate of multidrug-resistant pathogens is growing, meaning it is difficult to manage wounds effectively [16]. Consequently, the search for alternative treatments such as natural antibacterial clay therapy, hyperbaric oxygen therapy, therapeutic bacteriophages, antimicrobial peptides, cold plasma treatment, photodynamic therapy (PDT), honey, silver, bioelectric dressings, and photobiomodulation (PBM) has been ongoing, each with its own limitations [17,18]. This paper focuses on the effectiveness of PBM, with an emphasis on blue light, on wound management, and specifically on diabetic wounds.

## 2. Diabetic Wound Healing

**Figure 3:**
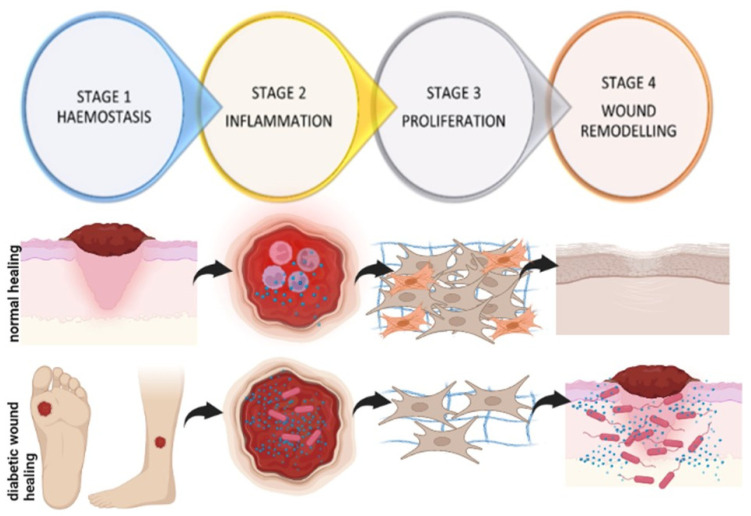
Sequence of events during wound healing. During haemostasis, there is the formation of a fibrin blood clot; this is soon followed by inflammation, which involves the infiltration of immune cells and inflammatory cytokines to remove pathogens and damaged tissue. During proliferation, there is migration and proliferation of different cell types, such as fibroblasts, cell differentiation (fibroblasts differentiate into myofibroblasts), and the synthesis and deposition of collagen. In the last phase of wound remodelling, there is scar tissue formation and maturation. In diabetic wound healing, wounds remain stalled in the inflammatory phase, with an increase in inflammatory cytokines, a decrease in immune cell infiltration, and a high risk of infection. There is decreased fibroblast migration, proliferation, and differentiation, and decreased collagen synthesis. Wounds frequently become chronic, extending for three months or longer and require an intensive treatment regime, often leading to lower limb amputation (created with BioRender).

Wound healing is a complex process involving multiple cell types, the extracellular matrix (ECM), and the action of soluble mediators such as growth factors and cytokines. Wound healing occurs as a cellular response to injury, which in turn leads to an activation of keratinocytes, fibroblasts, endothelial cells, macrophages, and platelets [19]. Many growth factors and cytokines released by these multiple cell types are needed to coordinate and maintain healing. Fibroblasts secrete numerous growth factors, cytokines, collagens, and other ECM components and therefore are fundamentally important in the tissue repair process, from the late inflammatory phase until the final epithelisation of the injured tissue [20,21,22,23,24,25]. The sequence of events of wound healing can be divided into four dynamic stages, (i) haemostasis, which involves coagulation and fibrin deposition, occurs immediately post-injury, and lasts for a few minutes; (ii) inflammation, which involves the removal of damaged tissue and pathogens and is characterised by neutrophils, macrophages and lymphocytes, and lasts for 4–6 days; (iii) proliferation, which involves the migration of different cell types, such as fibroblasts, and collagen deposition (2–10 days after injury); and (iv) wound remodelling with collagen cross-linking, scar tissue formation, and maturation [26,27,28,29,30] (Figure 3).

It is a well-known fact that diabetes impairs all the stages of wound healing [29]. Delayed diabetic wound healing is multifactorial but has been specifically associated with impaired cellular functioning and decreased cellular migration, proliferation, and the reduced synthesis of growth factors and collagen (Figure 3). There is also an increase in collagen-degrading enzymes (matrix metalloproteinases, MMPs). A prolonged inflammatory phase of wound healing and an increase in oxidative stress also leads to increased cell death [31]. It was established that wound healing is compromised, whereby cell proliferation and migration are affected, with a prolonged inflammatory phase of wound healing and increased pro-inflammatory markers such as interleukin (IL)-6 and tumour necrosis factor-alpha (TNF-α) in diabetic patients [32]. Skin biopsy analysis showed that diabetic patients have increased immune cell infiltration and increased expression of MMP-9 and protein tyrosine phosphatase-1B (PTP1B), which negatively regulates the signalling of insulin, leptin, and growth factors necessary for wound healing, leading to delayed healing [33]. Other factors such as reduced blood flow and infection further complicate the issue, meaning the treatment of such wounds is even more difficult.

There are several local and systemic management strategies that are necessary to implement to ensure wound closure. These include wound offloading, regular dressing changes that provide a moist wound environment, non-surgical or surgical debridement when necessary, antibiotic therapy with or without surgical intervention if osteomyelitis or soft tissue infection is present, optimal control of blood glucose, and the evaluation and correction of peripheral arterial insufficiency [34,35,36,37,38,39,40]. Infections in DFUs can be difficult to treat due to impaired microvascular circulation which leads to a limitation of phagocytic cells in the infected area and a decreased concentration of antibiotics in the infected tissues [41]. PBM has been shown to have successful benefits in wound closure under hyperglycaemic conditions, as well as bacterial eradication.

## 3. Wound Healing and Photobiomodulation (PBM)

PBM, formerly referred to as low-level laser (or light) therapy (LLLT), is a non-invasive, non-thermal treatment modality that involves the application of light (typically utilising lasers and light emitting diodes, LEDs) at certain wavelength spectra to living tissue and cells. The photon energy is absorbed by the cells and induces various photochemical and photophysical events. The part of the electromagnetic spectrum that is known as “light” ranges from UVC (200–280 nm), UVB (280–320 nm), UVA (320–400 nm), visible (400–750 nm), near infrared (NIR, 750–1200 nm) and mid/far IR (1200–10,000 nm) [42]. Red and NIR wavelengths have been shown to be beneficial, and more recent studies indicate that other wavelengths within the visible spectrum could be helpful as well, including blue light (400–500 nm). Research has identified that bacteria (Gram-positive, Gram-negative, mycobacteria), fungi (yeasts and filamentous fungi), viruses (DNA and RNA), and parasites can be effectively destroyed by light. Furthermore, the antimicrobial effectiveness seems to be unaffected by the antibiotic resistance of microbes, nor does it lead to resistant microbes after repeated sub-lethal light applications [42]. Irradiation with red or NIR light promotes tissue repair whilst blue light has been seen to take on a more antimicrobial function [43]. This could be beneficial in the fight against infected diabetic wounds.

### 3.1. The Effect of Red/Near-Infrared (NIR) Light: Mechanisms Involved

PBM related to the red and NIR spectra has been shown to be beneficial in treating diabetic ulcers which did not respond to conventional treatments [31]. Diabetic cells responded favourably to PBM in the wavelength range of 630 nm and 980 nm and at a fluence of between 1 J/cm^2^ to 5 J/cm^2^. These responses included an increase in the migration, viability, and proliferation of diabetic cells in vitro, as well as a stimulatory effect on the mitochondria with a resulting increase in adenosine triphosphate (ATP) [31]. Mitochondria are responsible for energy (ATP) production and are involved in cell regulation, signalling, and cell death. Mitochondria isolated from various fibroblast cell models (normal, diabetic, and ischemic) irradiated in vitro at a wavelength of 660 nm and a fluence of either 5 or 15 J/cm^2^ demonstrated a significant increase in ATP [44]. There also appeared to be a higher accumulation of active mitochondria in the irradiated groups. It was therefore concluded that irradiation with low-intensity visible red light (660 nm) can alter mitochondrial activity and increase ATP synthesis that modulates other cellular processes [44].

It is also known that visible red and NIR light interacts with photoreceptor molecules like cytochrome C oxidase (CCO) [44,45]. CCO (unit IV) forms part of the mitochondrial respiratory chain/electron transport chain (ETC) and transfers electrons from cytochrome c to molecular oxygen. It contains both heme (heme a and heme a3) and copper centres (CuA and CuB) capable of absorbing visible red and NIR light. It was first shown that the redox state of CCO is influenced by visible red light (632.8 nm) [46]. It was later highlighted that exposure to red and NIR (IR-A) light intensified the transfer of electrons in CCO, resulting in accelerated oxidative phosphorylation, hence ATP synthesis [47]. Other enzymes involved in the ETC have also been shown to respond to PBM using red and NIR light. An increase in enzyme kinetics was found in complex I (NADH ubiquinone oxidoreductase) and III (succinate dehydrogenase), as well as complex IV (CCO) in irradiated isolated mitochondria exposed to a wavelength of 660 nm (10 mW/cm^2^; 0.6, 1.2, 2.4, and 4.8 J/cm^2^) [48]. In a similar study also using a wavelength of 660 nm (11 mW/cm^2^, 5 J/cm^2^) there was an upregulation of genes coding for complex I, IV, and V [49]. It has also been shown that PBM causes the photo-dissociation of inhibitory nitric oxide (NO) bound to CCO, freeing the enzyme for oxygen binding, and leading to increased mitochondrial activity and ATP production [50].

Another hypothesis is that PBM activates cellular light-sensitive ion channels, allowing calcium ions (Ca^2+^) to enter the cell. Mitochondrial stimulation leads to increased intracellular reactive oxygen species (ROS) production, which in turn stimulates and activates numerous cell signalling pathways [50]. It has been stated that various wavelengths of light employed in PMB ranging from blue, green, red, and NIR light can modulate ROS production, which can lead to increased cell proliferation. Light applied to ‘healthy’ cells and tissue induces small increases in ROS production, whilst PBM can induce decreases in ROS production in inflamed tissue, thereby having a non-inflammatory effect on disease management [51]. Cyclic adenosine monophosphate (cAMP, derived from ATP), NO, ROS, and Ca^2+^ lead to the activation of transcription factors, resulting in increased gene expression and subsequent protein synthesis, increased cell migration and proliferation, and anti-apoptotic and anti-inflammatory effects [40]. Downstream intracellular responses are in response to photo-signal transduction and amplification in response to ATP, ROS, and NO changes [52].

A recent systematic review on the effects of PBM on human dermal fibroblasts revealed a lack of understanding of the cellular mechanisms affected by PBM [53]. The review showed that irradiation within the 600–1070 nm range has many positive effects related to the wound healing process. Positive effects were particularly observed in the areas of cellular viability, proliferation and migration, ATP, and mitochondrial function, as well as changes in protein and gene expression. The review further highlighted the importance of creating an optimal and widely accepted in vitro framework to ensure and improve the reliability and validity of results [53].

### 3.2. The Effect of Blue Light: Mechanisms Involved

Most of the literature on PBM focuses on wavelengths in the red and NIR spectrum (600–1100 nm), with fewer studies conducted on wavelengths less than 600 nm. Alternative therapies for the treatment of wounds have emerged, including the use the blue light (400–500 nm). For blue light to be an alternate or adjunct treatment option, it must enable cell proliferation and migration, which then could improve tissue regeneration. Alternatively, it could be used as an antimicrobial agent in combination with red and NIR light, thus providing both an antimicrobial and regenerative effect. However, such effects need to be studied.

Blue light has also been shown to affect mitochondrial activity and function. Earlier studies conducted by Karu [54] showed that blue light at 404 nm was absorbed by CCO, leading to cellular changes (including cell attachment and DNA and RNA synthesis). Blue light at 430 nm restored mitochondrial respiration which had been inhibited by NO and glycerol-trinitrate (GTN) [55], and blue light (400–450 nm) increased mitochondrial activity [51]. Opsonins (G-protein coupled receptors) exhibit an absorption spectrum ranging between 380–496 nm. Few studies have shown the effects of blue light on these receptors, and the mechanisms of these effects are still unclear [51]. Blue light can stimulate flavins and flavoproteins. Flavin mononucleotide (FMN), located within mitochondrial complex I and which catalyses the reduction in oxygen to superoxide, is activated by blue light, and hence leads to an increase in ROS [56,57]. Mitochondrial complex II, which contains flavin adenine dinucleotide (FAD) and is reduced to FADH2, has also been shown to absorb blue light [58].

### 3.3. Antibacterial Effects of Blue Light

Although the exact mechanism of the antimicrobial effects of blue light has not been fully uncovered, several biochemical processes resulting in cell death have been proposed. It has been suggested that the mechanism of bacterial cell death is probably photochemical rather than photothermal [59]. It has been proposed that blue light can be sensed by numerous microorganisms; consequently, it can regulate bacterial motility, suppress biofilm formation, and potentiate the light inactivation of bacteria [60]. The mechanism of blue light inactivation of *Cutibacterium acnes* (*C. acnes*) and *Helicobacter pylori* (*H. pylori*) and some oral bacteria was found to be due to the photoexcitation of intracellular porphyrins and the subsequent downstream production of cytotoxic ROS. The same was considered for wound pathogens such as *Staphylococcus aureus* (*S. aureus*) and *Pseudomonas aeruginosa* (*P. aeruginosa*), although this hypothesis has not been rigorously tested [60]. An in vivo study using blue light (445 nm) at 60 J/cm^2^ on a *P. aeruginosa* cutaneous rat wound infection model effectively decreased the bacterial load in young and established biofilms [59]. The results from bacterial cultures irradiated with one or two doses of 405 nm laser light (each consisting of 121 J/cm^2^) suggest that the initial antimicrobial activity of blue light alters cell membrane integrity, with a consequent decrease in membrane polarisation and rapid alteration of vital cellular functions [61]. While the wavelength range of 402–420 nm has been reported to be the most effective range, 455 nm and 470 nm have also been found to be of antimicrobial potential for some bacterial species (e.g., *S. aureus*). It was found that irradiation with blue light at 450 nm inhibited the growth of *S. aureus*, *Escherichia coli* (*E. coli*), and *P. aeruginosa* strains and was maintained for up to 48 h post irradiation [62]. Furthermore, there was no difference in bacterial growth between 24 h and 48 h, thus presenting no time-dependent effect. Moreover, no dose-dependent relationship was found.

A low energy density of 6 J/cm^2^ was found to be effective in inhibiting bacteria in vitro. Studies have irradiated *C. acnes* and Methicillin-resistant *Staphylococcus aureus* (MRSA), respectively, either in planktonic cultures, forming biofilms, or formed biofilms [63,64]. The results indicated that pulsed blue light at 450 nm and a 33% duty cycle performed at 3 h intervals using a low irradiance of 2 mW/cm^2^ and a fluence of 5 J/cm^2^ gave 100% suppression of *C. acnes*. However, due to the rapid rate of replication of MRSA, to achieve 100% bacterial suppression in planktonic cultures, it was found that irradiation needed to take place three times at 30 min intervals during a 24 h period at 3 mW/cm^2^ and 7.6 J/cm^2^ radiant exposure. The biofilm disruption of both bacteria was noted with the same irradiances and radiant exposures that gave 100% bacterial suppression in planktonic cultures, more so in forming biofilms than formed biofilms. However, it was noted that there was minimal bacterial suppression of each bacterium in forming and formed biofilms. The authors have suggested improving protocols with a combination of longer light exposure times, higher irradiance, and higher radiant exposures to achieve a more successful eradication of these bacteria in biofilms [63,64].

### 3.4. Effects of Blue Light on Fibroblasts and Wound Healing

In vitro studies published between 1999 and 2010, using multiple light sources, were limited, and had identified varying reports regarding the effects of blue light on mammalian cells, wound healing, as well as on antimicrobial efficacy and optimal therapeutic wavelengths [60]. These studies showed that blue light, under certain wavelength ranges and light exposures, may be toxic to various mammalian cells including keratinocytes, fibroblasts, retinal epithelial cells, and skin-derived endothelial cells. These studies also indicated that blue light caused damage to mammalian cells in a wavelength-dependent manner [60].

Irradiation with blue LED light using 412, 419, and 426 nm wavelengths at 66–100 J/cm^2^ and 453 nm at >500 J/cm^2^ was found to be cytotoxic to human keratinocytes and skin-derived endothelial cells [65]. The application of blue light from three common dental light sources, quartz–tungsten–halogen (QTH), plasma-arc (PAC), and laser, on the cellular function of 3T3 mouse fibroblasts in vitro, showed that exposures ranging from 5 J/cm^2^ (laser) to 15 J/cm^2^ (PAC and QTH) appeared to irreversibly suppress succinic dehydrogenase (SDH) mitochondrial activity up to 72 h post exposure. For the PAC and QTH sources, exposures as low as 3.5 J/cm^2^ also irreversibly suppressed SDH activity [66]. Blue light was reported to induce mitochondrial DNA damage and free radical production in human primary retinal epithelial (PRE) cells exposed to visible light (390–550 nm) at 2.8 mW/cm^2^. It was concluded that visible light can cause cell dysfunction through the action of ROS on DNA [67]. Blue light in several wavelength ranges filtered from a xenon arc lamp (400–410, 445–455, 450–490, or 485–495 nm) stimulated hydrogen peroxide (H_2_O_2_) production in cultured mouse fibroblasts, monkey epithelial cells, and human keratinocytes, thereby causing cellular damage when exposed to blue light [68]. On the other hand, narrow-band blue light (420 nm) applied at a radiant exposure of 54 mJ/cm^2^ and 134 mJ/cm^2^ showed that blue light had some anti-inflammatory effects on keratinocytes (HaCaT and hTERT) [69].

Similarly, studies after 2010 have identified varying results of effectiveness using PBM as an alternative to wound healing therapy [31]. This is particularly in in vivo studies, whereby, due to its stimulatory effect and no reported side effects, PBM has been used to treat chronic wounds. As mentioned previously, recent literature has highlighted that blue light in the range of 410–430 nm delivered to cultured cells in a dose range of 3.43–41.2 J/cm^2^ is able to modulate cell metabolism and proliferation. The response is different depending on cell types and it is dose-dependent. It has been identified that there are possible anti-proliferative effects when irradiated with higher doses in the wavelength range of 410 nm–430 nm, however, lower doses of blue light in this wavelength range appear to promote wound healing [70].

A comparison of the effects of blue and red LED light on in vivo wound healing in an excision wound model in rats found that irradiation at either 470 nm (blue) or 630 nm (red) with an intensity of 50 mW/cm^2^ (10 min for 5 consecutive days) substantially influenced wound healing [71]. The study also focused on the effects of PBM on gene expression and found that both wavelengths decreased keratin-1 mRNA, while keratin-10 mRNA levels were elevated in both light-treated groups compared to the control. This signifies that re-epithelialisation was not yet complete in both groups. Keratin-17 mRNA was also elevated in the red light group but was unchanged in the blue light group. This correlated to the fact that wound healing in the blue light group was nearly complete or that the wounds were already closed, while in the red light group, wound healing was incomplete. Keratins are important for the mechanical stability and integrity of epithelial cells and tissues. Keratin regulates intracellular signalling pathways that are involved in protection from stress and apoptosis and in wound healing. The study concluded that in contrast to previous studies, blue light significantly led to enhanced epithelialisation and decreased wound size and could play an important role in normotrophic wound healing by affecting keratin expression [71].

A 2008 study hypothesised that blue light influences normal human gingival fibroblast cells. Human gingival fibroblasts were exposed to single doses of halogen (186 J/cm^2^), LED (162 J/cm^2^), and plasma arc (240 J/cm^2^) irradiation for 240, 180, and 120 s, respectively. The result of the relatively high doses of irradiation indicated that blue light caused a mild inhibition of gingival fibroblasts’ proliferation after irradiation and further studies are recommended to clarify the exact mechanism underlying this effect [72].

In a study using Light Emitting Diode-Generated Blue Light (LED-BL), the effects of blue light at distinct wavelengths (410, 420, 453, and 480 nm) on the viability, proliferation, and antioxidative capacity of human dermal fibroblasts were observed [73]. It was shown that irradiation with blue light (410 and 420 nm) led to intracellular oxidative stress and toxic effects depending on the dose and wavelength. No toxicity was observed at 453 nm and 480 nm. Proliferating fibroblasts were seen to be more susceptible to blue light at 410, 420, and 453 nm, and at low doses of irradiation. There was a reduction in the antioxidative capacity of fibroblasts and a reduction in fibroblast proliferation. Different wavelengths caused varying degrees of intracellular oxidative stress with different physiological outcomes [73]. Similar observations were performed with 415 ± 15 nm LED-BL; the effect was dose-dependent (10, 15, 30, and 80 J/cm^2^) in terms of the significant inhibition of fibroblast proliferation, decreased cell numbers, and the increased generation of intracellular ROS. However, there was no significant effect on cell viability. Further observed was decreased fibroblast migration at energy densities of 5, 30, 45, and 80 J/cm^2^ [74]. This suggests that at these doses, this wavelength may not be favourable to fibroblast proliferation and wound closure. These antiproliferative and toxic properties of LED-BL, however, have been identified as possible alternative, safe, and cost-effective modalities for the prevention of fibrotic skin disease, hypertrophic scars, and the prevention of keloids [73,74].

In an in vitro scratch wound model, whereby human dermal fibroblasts were cultured for 48 h, fluorescence analysis showed viable cells regardless of irradiation doses within the range of 3 to 10 J/cm^2^ [43]. The study investigated the effect of 470 nm blue light on wound healing in terms of wound closure, total protein and collagen synthesis, and growth factor and cytokines, and concluded that irradiation did not impair in vitro wound healing. At 5 J/cm^2^ blue light appeared to promote protein synthesis [43]. The significant decrease in IL-6 suggests that light at 470 nm is anti-inflammatory. A subsequent study by the same authors using four assays of measurement to detect potential toxicity to fibroblasts irradiated with 470 nm blue light (3, 55, 110 and 220 J/cm^2^) revealed that the four assays differed in their levels of sensitivity to cell viability [75]. However, it was noted that with increasing doses, there was an alteration of mitochondrial metabolism, followed by lysosomal dysfunction, membrane disruption, and, finally, a loss of cell membrane integrity. It was found that irradiation with 3 J/cm^2^ or 55 J/cm^2^ did not adversely affect cell viability. However, the viability of human fibroblasts decreases progressively with increasing doses of 470 nm light, particularly at doses in the range of 110 J/cm^2^ or higher. Thereby concluding that doses below 110 J/cm^2^ appeared to be safe for fibroblast integrity [75].

Blue LED light (410–430 nm), using different light doses (3.43, 6.87, 13.7, 20.6, 30.9, and 41.2 J/cm^2^) on cultured HaCaT cells and human fibroblasts confirmed the capacity of blue light to modulate cell metabolism and proliferation. A reduction in HaCaT cell metabolism was observed in a dose-dependent manner 24 h post irradiation. A significant reduction in cell metabolism was observed when applying doses in the range of 20.6–41.2 J/cm^2^. Cell proliferation was not significantly affected by any of the doses. The irradiated fibroblasts, however, showed different responses to blue light depending on the dose. An increase in metabolic activity was observed with the application of 3.43 J/cm^2^, while doses of 20.6, 30.9, and 41.2 J/cm^2^ reduced cell metabolism 24 h post irradiation. This effect seemed to be more pronounced 48 h post irradiation. At 24 h post irradiation, a reduction in proliferation was observed at 41.2 J/cm^2^ [70].

A recent systematic review revealed that 72% of the literature reported beneficial therapeutic effects using blue light and 75% using green light [51]. The review found that blue and green light can modulate signalling pathways. It has been proposed that small increases in ROS production lead to an increase in cell proliferation, whilst a large increase can induce apoptotic signalling pathways. It has been established that blue light could produce oxidative stress in live mouse skin, preferentially in mitochondria, but green, red, far red, or infrared light did not [76]. It was further shown that blue light induced oxidative stress in cultured human keratinocytes. Exposing human skin to the blue light contained in sunlight depressed Flavin autofluorescence. It has been suggested that blue light could contribute to the same biological effects (skin ageing and an increase in pigmentation) as UVA radiation because wavelengths of blue light are closely related to the UVA spectrum [76,77]. However, it has been stated in some earlier studies that visible blue light does not cause DNA damage or early photo-ageing and that the use thereof in dermatological practice was safe (420 nm at 20 J/cm^2^ on five consecutive days) [77]. Recent studies, however, have shown that visible blue light (450–465 nm at a power density of 42.05 mW/cm^2^) at higher fluences (38 J/cm^2^) has a negative impact on cellular morphology, hyperpigmentation, mitochondrial hyperpolarisation, and oxidative stress [78]. Blue light at a fluence of 58 ± 20 J/cm^2^ induced pigmentation in darker skin types (skin type III and IV) for longer periods of time [79,80].

Opsonins have been found in skin and may function as photoacceptors. Opsin 3 (OPN3, encephalopsin) has been found to be activated in response to low-dose blue light (453 nm, 3.2 J/cm^2^) in hair keratinocytes, where it increased cellular proliferation and exerted a positive effect on hair growth ex vivo [81]. The activation of OPN4 (melanopsin) in response to blue light (~430 and 460 nm) in mice resulted in vasodilation [82]. Castellano-Pellicena and colleagues investigated the effect of blue light (453 nm) at 2 J/cm^2^ on opsonins in an ex vivo wound model and an in vitro scratch-wound assay [83]. It was found that blue light stimulated keratinocyte differentiation (ex vivo), and this was linked to OPN3. Blue light had no effect on keratinocyte morphology and migration (scratch-wound assay) but did cause a decrease in DNA synthesis, and at a higher fluence of 30 J/cm^2^, migration was inhibited [83]. It has been cited that the blue light activation of OPN3 leads to tyrosinase/tyrosinase-related protein complex formation and may increase melanogenesis in skin types [79,80]. The studies used in this review are summarised in Table 1.

Wavelength/s selection is frequently based on tissue penetration depth, whereby longer wavelengths penetrate tissue more deeply. Combination treatments with different wavelengths for optimal wound management have been suggested. It is often suggested that the most optimal wavelengths for wound healing treatments are those that have the greatest depth of penetration inside tissue. It has been concluded by some that the optimal wavelength for wound closure is in the red region (730 nm), whilst blue light (480 nm), which has an estimated ~0.5–1 mm penetration depth, is said to be optimal for the treatment of infected wounds [80,84]. Blue light does not penetrate as deeply as red or NIR light and may not reach deeper infected tissue. Being able to target tissues at depth should be considered together with delivering the right amount of energy to the target tissue over the correct time duration [85]. Variations in tissue types have produced different results when it comes to tissue penetration depth, and variations in melanin concentrations and tissue water have led to different effects [86]. There does not seem to be a simple answer for light dosing and tissue penetration to achieve optimal outcomes for PBM regimes [85].

## 4. Conclusions

Diabetes is a severe life-threatening disease of global proportions. South Africa has the highest prevalence of diabetes (4.2 million) and diabetes-attributed mortality rate (97,676) in Africa. South Africa is also not the only developing country faced with high prevalence rates and the severity of diabetes-related complications such as amputations, disability, and quality of life issues. Considering the projected increase in diabetic cases, diabetic foot-related complications, the reality of multi-drug resistant pathogens, financial burdens, decreased quality of life and numerous comorbidities that are associated with diabetes, and the ever-increasing need for limb salvage, the urgency of finding holistic, multi-factorial, alternate, and/or adjunct therapies such as PBM is imperative.

PBM at different wavelengths has shown positive results with regards to wound healing and fibroblast proliferation. It is also worth taking into consideration that red and NIR wavelengths have established beneficial effects in diabetic ulcer care. Recent studies indicate that other wavelengths within the visible spectrum could be beneficial as well, including blue light (400–500 nm). Not all studies involving the use of blue light have shown positive effects; however, those that have noted that there must be a careful selection of blue light wavelength and dose. In general, it has been observed that low doses of blue light appear to promote wound healing. High doses of blue light, above 50 J/cm^2^, appear to impair cell proliferation, particularly in the 410 nm to 430 nm wavelength range. This may still be beneficial in reducing fibrotic skin disease and preventing hypertrophic scar and keloid formation.

Even with the current plethora of knowledge regarding blue light irradiation on the effect of fibroblasts and wound healing, no study has identified the silver bullet of wavelengths and dosing regimens for optimum fibroblast proliferation and wound healing. Given the antibacterial effects of blue light on drug-resistant bacteria, the study of blue light on fibroblasts and diabetic wound healing, alone or in combination with other wavelengths, is warranted. The mechanisms of the bactericidal effect of blue light as well as the effects of blue light on mammalian cells are not yet fully understood; however, the literature of late is showing promising therapeutic benefits.

Blue light is ubiquitous and relatively inexpensive; it is available in the form of LEDs. Therefore, blue light could be an easily applicable, safe, and cost-effective treatment of surface wounds.

Further studies need to determine the optimum wavelengths and fluences on infected cultures, as currently, many studies performed in vitro primarily involve either bacteria or cells alone. Research has advanced to a point whereby clinical trials will be able to strengthen the evidence. In vivo studies are necessary to explore what has been identified as effective in vitro protocols with an added consideration of other factors such as blood glucose levels, vascular status, environmental factors, types of dressings, and how all these may affect the dosage regimes and effectiveness of light therapy.

## Figures and Tables

**Table 1:** Summary of the effects of photobiomodulation (PBM) at various wavelengths on wound healing.

Cell/Species Type	Irradiation Parameters	Study Outcomes/Results	Author Reference
Human fibroblasts (WS1) modelled into wounded, diabetic wounded, and ischemic cell models	Continuous wave diode laser at 660 nm with 5 J/cm^2^. Control cells received no laser irradiation. Cells were incubated at 37 °C for 30 min post-irradiation.	Upregulation of genes encoding for subunits involved in mitochondrial electron chain complexes I (NADH: ubiquinone Oxidoreductase), IV (cytochrome c oxidase), and V (ATP synthase).	Masha et al. [49]
Human fibroblasts (WS1) modelled into wounded, diabetic wounded, and ischemic cell models	Mitochondria were isolated from various cell models and irradiated at 660 nm with either 5 or 15 J/cm^2^. Non-irradiated mitochondria served as controls.	Alteration of mitochondrial function, in particular an increase in cytochrome c oxidase (CCO) activity, as well as an increase in adenosine triphosphate (ATP) synthesis.	Houreld et al. [44]
HeLa cells	Monochromatic radiation in the range of wavelength 600–860 nm, with a light intensity of 1.3 W/m^2^, dose 52 J/m^2^, and irradiation time of 40 s.	Visible red and near-infrared light interacts with photoreceptor molecules like CCO.	Karu et al. [45]
HeLa cells	Cells were irradiated three times at 632.8 nm, fluence of 6.3 × 10^3^ J/m^2^ for 10 s.	The redox state of CCO is influenced by visible red light.	Karu et al. [46]
Sprague Dawley Rat, liver mitochondria	Argon-dye laser at a wavelength of 660 nm, power density of 10 mW/cm, at fluences of 0.6 J/cm^2^, 1.2 J/cm^2^, 1.8 J/cm^2^, 2.4 J/cm^2^, and 4.8 J/cm^2^. Experimental fluences were achieved by varying the irradiation times (1, 2, 3, 4, or 8 min).	An increase in enzyme kinetics was found in complex I (NADH ubiquinone oxidoreductase) at fluences of 1.2 J/cm^2^ and 2.4 J/cm^2^ and complex III (succinate dehydrogenase) and complex IV (CCO) at fluences of 0.6 J/cm^2^, 1.2 J/cm^2^, 2.4 J/cm^2^, and 4.8 J/cm^2^.	Yu et al. [48]
Sprague Dawley Rat, liver mitochondria	Lumination of samples using light emitting diodes (LED) at 629 nm (red), 1 W, 44 lumen; 530 nm (green), 1 W, 30 lumen; 470 nm (blue), 1 W, 10 lumen. Irradiance intensity of 50 mW/cm^2^.	Mitochondrial respiration inhibited by nitric oxide (NO) and glycerol-trinitrate (GTN) was completely restored by illumination at 430 nm (blue light).	Dungal et al. [55]
HeLa cells	Blue light (462 nm) irradiation at a fluence of 3.744 J/cm^2^, frequency of 0.52 mW/cm^2^, irradiated for 2 h.	Blue light triggers the cytotoxicity of riboflavins in HeLa cells.	Yang et al. [56]
Human oral mucosa epithelial cells Human skin keratinocytes Bacteria (*Pseudomonas aeruginosa*)	Blue light (445 nm) at three different protocols: Protocol A: ≤0.30 W/cm^2^, at 40 J/cm^2^ (low), 60 J/cm^2^ (intermediate), and 120 J/cm^2^ (high); Protocol B: 0.31–0.60 W/cm^2^, at 40 J/cm^2^ (low), 60 J/cm^2^ (intermediate), and 120 J/cm^2^ (high); Protocol C: ≥0.61 W/cm^2^, at 40 J/cm^2^ (low), 60 J/cm^2^ (intermediate), and 120 J/cm^2^ (high).	All protocols delivering blue laser light effectively reduced bacterial growth at 24 h. The antimicrobial activity of blue laser light on *P. aeruginosa* relies on the generation of oxidative stress with minimal toxicity to mammalian cells and tissues.	Rupel et al. [59]
Human keratinocytes and skin-derived endothelial cells	Light-emitting diodes (LED) irradiation at: 412 nm (0, 33, 66, and 100 J/cm^2^ at 87 mW/cm^2^), 419 nm (0, 33, 66, and 100 J/cm^2^ at 126 mW/cm^2)^, 426 nm (0, 33, 66, and 100 J/cm^2^ at 68 mW/cm^2^), 453 nm (0, 33, 66, and 100 J/cm^2^ at 66 mW/cm^2^), 632 nm (0, 20, 40, and 60 J/cm^2^ at 38 mW/cm^2^), 648 nm (0, 20, 60, and 100 J/cm^2^ at 71 mW/cm^2^), 850 nm (0, 60, and 120 J/cm^2^ at 50 mW/cm^2^), and 940 nm (0, 40, and 80 J/cm^2^ at 32 mW/cm^2^). UVA irradiation, using a mercury arc lamp unit, emitting a UVA spectrum (340–410 nm) with a maximum intensity of 366 nm (84 mW/cm^2^).	Blue light irradiation with 412–426 nm at high fluences is toxic for endothelial cells and keratinocytes thereby reducing cell numbers. Blue light irradiation with 453 nm is not toxic at high fluences but reduces cell proliferation dose-dependently. Neither red (632–648 nm) nor infrared (850–940 nm) irradiation caused significant changes in cell proliferation.	Liebmann et al. [65]
3T3 Mouse fibroblasts	Blue light using Quartz–tungsten–halogen (QTH), plasma-arc (PAC), and laser at fluences including 1, 3, 6, 10, 15, 20, 30, and 60 J/cm^2^. Intensities of 556 mW/cm^2^ (QTH), 1690 mW/cm^2^ (PAC), and 202 mW/cm^2^ (laser) were used. The maximum time for the QTH light source was 120 s (60 J/cm^2^), based on three 40 s curing cycles. The PAC source was used for up to 30 s (60 J/cm^2^), and the laser was used for up to 20 s (5 J/cm^2^).	Suppression of succinic dehydrogenase (SDH) activity of mitochondria ranging from 5 J/cm^2^ (laser) to 15 J/cm^2^ (PAC, QTH) up to 72 h post-exposure. Significant suppression of SDH activity at 1 J/cm^2^ for the PAC source but no suppression was noted for the laser and QTH source. At 3.5 J/cm^2^, SDH activity suppression was seen in the PAC and QTH sources. Temperature rises ranged from 2 to 9 °C above the base temperature of 37 °C. The cellular effects did not appear to be caused by increases in temperature alone, and the effects were light-dose-dependent.	Wataha et al. [66]
Human primary retinal epithelial cells	Blue light at 390–550 nm and 2.8 mW/cm^2^ for 0–9 h.	There was no significant difference in mitochondrial respiration detected at 3 h as compared to controls. However, a small decrease in cell viability was observed at 6 h in irradiated cells. At 9 h cell viability decreased even further.	Godley et al. [67]
Mouse fibroblasts (NIH 3T3 cells), African green monkey kidney epithelial cells (CV1 cells), and human foreskin keratinocytes (HK cells)	Cells irradiated using a 75 W Xenon Arc Lamp with interference filters at various wavelengths: UVA (375–385 nm), violet (400–410 nm), violet-blue (445–455 nm), blue (450–490 or 485–495 nm), green (495–505 or 500–560 nm), orange (550 long-pass filter), and red (590–650 nm or 605 long-pass filter).	Violet-blue light- and UVA-stimulated hydrogen peroxide production in cultured mouse fibroblast, monkey kidney, and human keratinocytes leading to cellular damage.	Hockberger et al. [68]
Human skin keratinocytes (HaCaT and hTERT)	Cells were treated with interferon-gamma (INF-γ) and tumour necrosis-alpha (TNF-α) and irradiated with UVB (312 nm at 50 mJ/cm^2^) and/or blue light (420 nm at 54 mJ/cm^2^ and 134 mJ/cm^2^).	Blue light and low-dose UVB treatment of HaCaT and hTERT cells resulted in the inhibition of cytokine-induced production of interleukin (IL)-1α. Exposures to 54 mJ/cm^2^ and 134 mJ/cm^2^ showed that blue light had some anti-inflammatory effects.	Shnitkand et al. [69]
Human keratinocyte (HaCaT), and Human healthy skin samples and fibroblasts	Blue LED (420 nm) at 3.43, 6.87, 13.7, 20.6, 30.9, and 41.2 J/cm^2^. Power density of 680 mW/cm^2^ and varying irradiation times from 5 to 60 s.	Blue LED light (410–430 nm) in the range of 3.43–41.2 J/cm^2^ can modulate metabolism and proliferation in healthy human cells in a dose and cell-dependent manner.	Rossi et al. [70]
Human gingival fibroblasts	Blue light: Halogen at 750 mW/cm^2^ and 186 J/cm^2^ for 240 s. LED 900 mW/cm^2^ and 162 J/cm^2^ for 180 s. Plasma arc irradiation 2000 mW/cm^2^ and 240 J/cm^2^ for 120 s.	All types of blue light irradiation have led to diminished cell proliferation by 40% one-week post exposure and were not attributed to the formation of DNA double-strand breaks and cannot be annulled by N-acetyl-cysteine.	Taoufik et al. [72]
Human dermal fibroblasts (HDF)	Blue LED light at 410, 420, 453, 480 nm with 0 J/cm^2^, 15 J/cm^2^, 30 J/cm^2^, 60 J/cm^2^, 90 J/cm^2^. Power density of 50 mW/cm^2^.	Blue LED light causes toxicity and reduced proliferation in human dermal fibroblasts in a dose and wavelength-dependent manner. Toxicity was identified at 410 nm (60 J/cm^2^) and 420 nm (60 J/cm^2^ and 90 J/cm^2^). There was an increase in intracellular oxidative stress in a wavelength-dependent manner (410 nm and 420 nm). Blue LED light also led to an increase in the sensitivity of human dermal fibroblasts to hydrogen peroxide.	Opländer et al. [73]
Human skin fibroblasts	LED-generated blue light (LED-BL) with 415 ± 15 nm at 350 W/m^2^ (0, 5, 10, 15, 30, 45, and 80 J/cm^2^).	LED-BL (415 nm) inhibits fibroblast proliferation in a dose-dependent manner without causing significant effects on viability at fluences of 10, 15, 30, or 80 J/cm^2^. Irradiation with fluences of 5, 30, 45, and 80 J/cm^2^ decreased fibroblast migration speed, and fluences of 5, 10, 30, and 80 J/cm^2^ resulted in an increase in reactive oxygen species.	Mamalis et al. [74]
Human skin fibroblasts	Blue light at 470 nm (30 mW/cm^2^). Cells were irradiated with a fluence of 3, 55, 110, and 220 J/cm^2^ and incubated for 24 h.	It was found that the MTT and Trypan Blue assay identified a significant decrease in cell viability when irradiated with 55, 110, and 220 J/cm^2^. At 3, 55, 110, and 220 J/cm^2^ the live/dead fluorescence assay identified only a slight decrease in cell viability. The neutral red assay identified a significant decrease in cell viability with 220 J/cm. Irradiation with 3 J/cm^2^ or 55 J/cm^2^ did not adversely affect cell viability. Thus, doses below 110 J/cm^2^ appear safe. As the dose increased, there appeared to be an alteration in mitochondrial metabolism, followed by lysosomal dysfunction, membrane disruption, and the eventual loss of cell membrane integrity.	Masson-Meyers et al. [75]
Mouse aortas	Irradiation doses were delivered via cold light lamp (Opelco 20500/06) (40,000–190,000 lux), light diodes [red (620–750 nm), green (495–570 nm), or blue (380–495 nm)] or a monochromator with varying wavelengths.	OPN4 mediates photorelaxation in blood vessels. Vasorelaxation is wavelength-specific, with a maximal response at ~430–460 nm.	Sikka et al. [81]
Human Keratinocytes Additionally, human hair follicles	LED-based device, hair follicles were irradiated with 453 or 689 nm wavelengths, 16 mW/cm^2^ irradiance (3.2 J/cm^2^) radiant exposures during 10 consecutive days. Cell monolayers were treated with 3.2 J/cm^2^ light (453 nm)	The expression of OPN2 and OPN3 was detected in skin and hair follicles. Treatment with 3.2 J/cm^2^ of blue light with 453 nm was seen to sustain cell proliferation of the outer root sheath cells and thereby have a positive on hair growth ex vivo.	Buscone et al. [82]
Human keratinocytes and dermal fibroblasts Human ex vivo: epithelial tongue cells	LED-based devices at 447, 505, 530, 655, and 850 nm wavelengths in vitro. Ex vivo wounds were irradiated daily with two proprietary LED devices emitting 453 nm light at 2 J/cm^2^ or 656 nm at 30 J/cm^2^	Blue light stimulated wound closure, with a corresponding increase in OPN3 expression. Blue light had no effect on keratinocyte morphology and migration (scratch-wound assay) but did cause a decrease in DNA synthesis, and at a higher fluence of 30 J/cm^2^, migration was inhibited.	Castellano-Pellicena [83]
Bacteria (methicillin-resistant *Staphylococcus aureus*; MRSA)	Violet/blue visible diode laser (405 nm), fluence of 121 J/cm^2^, power density of 135 mW/cm^2^, irradiated for 15 min. One or two irradiations.	Blue light rapidly suppresses MRSA by the alteration of membrane integrity with a decrease in membrane polarisation and alteration of vital cellular functions. MRSA activity is suppressed 5 min after the first dose and continues after the second dose. Two doses of blue light administered 30 min apart are more effective in reducing the number of viable cells than a single dose.	Biener et al. [61]
Bacteria (*Staphylococcus aureus*, *Escherichia coli*, and *Pseudomonas aeruginosa*)	A single blue laser irradiation (450 nm) at fluences of 0 (control), 3, 6, 12, 18, and 24 J/cm^2^.	*S. aureus* and *P. aeruginosa* inhibition at low fluences (>6 J/cm^2^), maintained for up to 48 h post irradiation, with no dose-dependent relationships noted. *E. coli* was inhibited at all fluences except at 24 J/cm^2^.	De Sousa et al. [62]
Bacteria (*Cutibacterium acnes*)	Bacteria irradiated three times per day at 3 or 4 h intervals over three or more days using a pulsed laser (450 nm) with fluencies of 3 or 5 J/cm^2^ and irradiance at 2 mW/cm^2^. *C. acnes* fluorescence intensity was measured at decreasing radiant exposures of 5, 3.6, and 3 J/cm^2^ on days one, two and three; and then 5 and 3.6 J/cm^2^ on day four at 2 mW/cm^2^.	Total (100%) bacterial suppression is achievable using 5 J/cm^2^ when applied three times per day at 3 h intervals over a three-day period. *C. acnes* fluoresce predominantly in the red wavelength range and diminish progressively with repeated irradiation at 3 h intervals; however, a resurgence of bacterial growth after long periods of no treatment was noted.	Bumah et al. [63]
Bacteria (*Cutibacterium acnes* and methicillin-resistant *Staphylococcus aureus*) in planktonic cultures, forming biofilms or formed biofilms	Planktonic bacteria cultures: MRSA irradiated at 450 nm (3 mW/cm^2^), with 0, 4.5, 5.4, or 7.6 J/cm^2^ three times at 30 min intervals; *C. acnes* cultures irradiated with 2 mW/cm^2^, 0, 3.6 or 5 J/cm^2^ thrice daily for three days at 3 h intervals. Forming biofilms irradiated at 450 nm (2 mW/cm^2^) with 0 or 7.6 J/cm^2^ three times per day for three days. MRSA irradiated at 30 min intervals and *P. acnes* irradiated at 3 h intervals. Established biofilms of MRSA and *C. acnes* were irradiated with pulsed light at 450 nm (2 mW/cm^2^) three times a day for 3 days either at 7.6 J/cm^2^ or 10.8 J/cm^2^.	Total (100%) bacterial suppression in planktonic cultures of MRSA and *C. acnes* with 7.6 J/cm^2^ and 5 J/cm^2^, respectively. There was no significant decrease in both bacteria in terms of the rate of biofilm formation, and the antimicrobial effects in forming and formed biofilms were minimal. However, increasing the radiant exposure to 10.8 J/cm^2^ yielded more disruption of the biofilm and fewer live MRSA and *C*. *acnes* were noted.	Bumah et al. [64]
Sprague Dawley Rat, infected excision wound	Blue Light (445 nm), at ≤0.30 W/cm^2^ and 60 J/cm^2^. Irradiation was performed at 30 min or at 24 h after infection with *P. aeruginosa*.	The inhibition of the progression of wound superinfection through intracellular ROS production.	Rupel et al. [59]
Sprague Dawley Rat, excision wound	Group 1 was treated with blue LED (470 nm, 1 W), Group 2 was treated with red LED (629 nm, 1 W), and Group 3 was not illuminated (control). For each light source, irradiance was 50 mW/cm^2^, and irradiation took place post-operatively and on five consecutive days for 10 min.	Blue light significantly reduced wound size by 50% on day 7 post-operatively. There appeared to be enhanced epithelisation. Both wavelengths also affected keratin mRNA expression.	Adamskaya et al. [71]
Hairless mice expressing roGFP1 Human live skin Human keratinocyte cells (HaCaT)	Mice irradiated with high-power LED-emitting UVA (365 nm), blue (460 nm), green (523 nm), red (623 nm), far red (740 nm), or infrared (850 nm). Mouse autofluorescence was recorded every 10 s. After 5 min of baseline recording, the skin was exposed to the LED light for 5 s (duty cycle 50%) between each fluorescence recording. HaCaT cells were irradiated with UVA (365 nm), blue (460 nm), and green (523 nm) light LED. Fluorescence ratios were recorded every 15 s, and cells were exposed to LED light for 7.5 s in between ratio recordings (duty cycle 50%). Human skin (left and right hands) irradiated with blue light equivalent to the blue component of direct sunlight. The average irradiance was 11 mW cm^2^ and corresponds to the high energy blue light component (wavelengths 400–480 nm). Human skin autofluorescence was recorded every 10 s. After 5 min baseline, blue light illumination was initiated at 80% duty cycle (8 s of illumination at 460 nm and 13.8 mW cm^2^ every 10 s) for 10 min, followed by another 5 min of autofluorescence recording without blue light illumination.	Blue light could produce oxidative stress in live skin, preferentially in mitochondria, but green, red, far red, or infrared light did not. Blue light-induced oxidative stress was also detected in cultured human keratinocytes. Skin autofluorescence was reduced by blue light, suggesting flavins are the photosensitiser. Exposing human skin to the blue light contained in sunlight depressed flavin autofluorescence, demonstrating that the visible component of sunlight has a physiologically significant effect on human skin. Blue light contributes to skin ageing similar to UVA.	Nakashima et al. [76]
Human live skin	Photodynamic therapy (PDT) lamp with an emission spectrum between 380 and 480 nm and peak emission at 420 nm. Irradiation was given on five consecutive days with 20 J/cm^2^, with a cumulative dose of 100 J/cm^2^.	No inflammatory cells and sunburn cells were visible before or after irradiation. However, there was an increase in the perinuclear vacuolisation of keratinocytes after 48 h. Irradiation does not cause DNA damage or early photo-ageing. Minimal hyperpigmentation of the irradiated skin was seen.	Kleinpenning et al. [77]

## Data Availability

Not applicable.
